# Menstrual cycle changes and mental health states of women hospitalized due to COVID-19

**DOI:** 10.1371/journal.pone.0270658

**Published:** 2022-06-24

**Authors:** R. Muharam, Feranindhya Agiananda, Yuri Fitri Budiman, Juliana Sari Harahap, Kevin Ardito Prabowo, Mazaya Azyati, Yuannita Ika Putri, Gita Pratama, Kanadi Sumapraja

**Affiliations:** 1 Faculty of Medicine University of Indonesia–Cipto Mangunkusumo National Hospital, Division of Reproductive Immunoendocrinology, Department of Obstetrics & Gynecology, Jakarta, Indonesia; 2 Faculty of Medicine University of Indonesia–Cipto Mangunkusumo National Hospital, Division of Consultant Liaison Psychiatry, Department of Psychiatry, Jakarta, Indonesia; 3 Faculty of Medicine University of Indonesia–Cipto Mangunkusumo National Hospital, Department of Psychiatry, Jakarta, Indonesia; 4 Faculty of Medicine University of Indonesia–Cipto Mangunkusumo National Hospital, Department of Obstetrics & Gynecology, Jakarta, Indonesia; Fondazione IRCCS Ca’ Granda Ospedale Maggiore Policlinico, ITALY

## Abstract

**Purpose:**

Many studies have evaluated the impact of the COVID-19 pandemic on women’s mental health and menstrual changes. However, most of these studies only included nonhospitalized COVID-19 patients, while information on hospitalized women is very limited. Thus, this study aimed to examine the mental health status and menstrual changes in hospitalized female COVID-19 patients.

**Methods:**

A survey was administered to female COVID-19 patients in the isolation ward of a national referral hospital in Indonesia between January and August 2021, and the women were followed up 3 months after discharge. The survey evaluated menstrual patterns and mental health using the Self Reporting Questionnaire-29 (SRQ-29).

**Results:**

The study enrolled 158 female patients. There was an increase in patients who had a cycle length of > 32 or < 24 days, and significant increases in menstrual irregularity and heavy menstrual bleeding were noted. Overall, 37.3% of the patients reported a change in menstrual pattern after infection with COVID-19. Based on SRQ-29 scores, 32.3% of the women had neurotic symptoms, 12.7% had psychotic symptoms, and 38.0% had symptoms of posttraumatic stress disorder. Patients with symptoms of mental health disorders were twice as likely to report a menstrual change (OR 2.17, 95% CI 1.12–4.22; p = 0.021).

**Conclusion:**

Menstrual changes and increased symptoms of mental health disorders occur in hospitalized female COVID-19 patients. The length of isolation was the key factor affecting overall menstrual changes and mental health in hospitalized female COVID-19 patients.

## Introduction

The COVID-19 pandemic, declared in March 2020 by the World Health Organization (WHO) [1], has continued globally for two years. The SARS-CoV-2 virus, which causes COVID-19, is transmitted through small liquid particles from an infected person’s mouth or nose when coughing, sneezing, speaking, singing, or breathing. These particles range from larger respiratory droplets to smaller aerosols [1,2]. Therefore, infected people need to be isolated from other people. In addition, infected people with moderate-to-severe symptoms need to be hospitalized in isolation rooms [3].

Patients in isolation wards have been reported to have a poorer therapeutic relationship with their health care providers than those who are not isolated. Health care teams spend less time with isolated patients and are always dressed in complete personal protective equipment, causing patients to feel that their care is depersonalized [4,5]. In addition, various stressors during isolation, such as fear of death, loss of social contact, loneliness, and financial loss, may significantly affect mental health [6,7].

A study found that the COVID-19 pandemic was significantly associated with higher levels of stress, anxiety, depression, posttraumatic stress disorder (PTSD), and more severe psychological impacts in women [8]. It is known that psychological distress can affect menstruation [9]. A study also showed that the degree of stress and anxiety caused by the COVID-19 pandemic was found to be high enough to affect the characteristics of the menstrual cycle in women [10].

Psychological distress is associated with menstrual changes. A study showed that depression and anxiety disorders affect the regulation of the hypothalamic–pituitary–adrenal (HPA) axis, which can inhibit the luteinizing hormone (LH) surge and lead to ovarian dysfunction [11]. Stress can affect the hypothalamic-pituitary-gonadal axis, altering the regulation of gonadotropin-releasing hormone, gonadotrophs, and the gonads [12]. In distress, the body produces cortisol, which can inhibit gonadotropin-releasing hormone (GnRH) secretion. Decreased GnRH secretion leads to reduced follicle stimulating hormone (FSH) levels, LH levels, follicular development, and estrogen secretion [13]. These changes can lead to anovulation and functional hypothalamic amenorrhea. Psychological distress has also been associated with worsening dysmenorrhea and heavy menstrual bleeding (HMB) [14,15].

The effects of COVID-19 on the reproductive system are still widely studied. SARS-CoV-2 infection could affect the hypothalamic-pituitary-ovarian-endometrial axis, resulting in menstrual cycle changes. Hypothalamic hypogonadism may occur in severe COVID-19, which can cause temporary amenorrhea and infrequent menstruation [16]. ACE-2 receptors are widely expressed in the ovaries and endometrium [17,18]. This could allow SARS-CoV-2 infection to directly affect ovarian hormones and endometrial responses that lead to menstrual disturbances [19]. Menstrual changes have also been reported after mRNA and adenovirus vectored COVID-19 vaccines are received. The mechanism behind this is immunological influences on menstrual cycle hormones [20]. The current study showed that changes in the menstrual cycle do occur following COVID-19 vaccination; however, the changes are slight and are reversed quickly [21].

Many studies have evaluated the impact of the COVID-19 pandemic and quarantine on women’s mental health and menstrual changes. However, most of them only included the general public, while information on hospitalized women is very limited. Therefore, this study aimed to investigate the mental health status and menstrual changes in hospitalized female COVID-19 patients.

## Methods

### Study design

We conducted an observational study using a cross-sectional approach. Two surveys were conducted in this study. The first survey was administered to female COVID-19 patients in the isolation ward of Cipto Mangunkusumo Hospital, Jakarta, Indonesia. A digital questionnaire was created using Google Forms (Alphabet Inc., Menlo Park, CA) and then distributed to patients using a tablet computer. The second survey was a follow-up questionnaire administered by researchers. The patients were asked to complete the digital questionnaire again via WhatsApp messenger (Meta, Menlo Park, CA) 3 months after discharge from the isolation ward. The study was conducted from January 2021 to August 2021.

The questionnaire consisted of three sections: 1) identity and demographic questions; 2) reproductive health history and menstrual patterns; and 3) the Self Reporting Questionnaire-29 (SRQ-29) (Appendix 1 in S1 Appendix). In the first survey, patients were asked to self-report their last menstrual pattern before being infected with COVID-19. In the second survey (3 months after discharge), patients were asked to report their present menstrual cycle and complete the SRQ-29. Patients were asked to include the start and end dates of their period to assess menstrual cycle length. The SRQ-29 was used to screen for symptoms of mental health disorders and has been regularly used by the Ministry of Health of the Republic of Indonesia for mental health screening [22].

Menstrual cycle length was divided into three categories according to the typical menstrual cycle in Asian women: shorter (< 24 days), normal (24–32 days), and longer (> 32 days). Twenty-four to 32 days is considered the normal/typical cycle length [23]. An irregular menstrual cycle was defined as a difference of ≥ 7 days in cycle lengths between months. HMB was defined as menstrual bleeding lasting more than 7 days in one cycle.

The SRQ-29 consists of 29 questions with ’yes’ or ’no’ answers. Of these 29 questions, questions 1–20 are related to neurotic symptoms (considered positive if a patient answers ‘yes’ to ≥ 5 questions), question 21 is related to psychoactive substance use, questions 22–24 are related to psychotic symptoms (considered positive if a patient answers ‘yes’ to ≥ 1 question), and questions 25–29 are related to posttraumatic stress disorder (PTSD) (considered positive if a patient answers ‘yes’ to ≥ 1 question).

### Ethical consideration

Ethical approval was granted by the Health Research Ethics Committee of the University of Indonesia and Cipto Mangunkusumo Hospital (ID: KET-1298/UN2.F1/ETIK/PPM.00.02/2020). All participants provided written informed consent. The raw data collected in this study are stored in computer files that can be accessed only by the researchers. The identities of the patients included in this study are represented with initials to maintain confidentiality.

### Study sample

A consecutive sampling method was used in this study. The study only included women of reproductive age (20–50 years). Pregnancy, hormonal contraceptive use, and amenorrhea due to any reason (intrauterine device, menopause, lactating, etc.) were the exclusion criteria of the study. A total of 187 patients participated in the study. However, 29 participants were eliminated because of a loss to follow-up after discharge. Ultimately, 158 patients completed the survey and were included in the analysis.

### Statistical analysis

Normally distributed data are reported as the mean and standard deviation, while skewed data are reported as the median and interquartile range. Student’s t test, or an equivalent nonparametric test, was conducted to analyze the differences in menstrual patterns before and after COVID-19 infection. McNemar’s test was used to analyze the difference in menstrual patterns before and after COVID-19 infection. Multivariate regression analysis was conducted if appropriate to determine which independent variables had the most impact on the dependent variable. The Pearson correlation coefficient, or Spearman’s rho where appropriate, was used to analyze the correlation between two variables. Statistical significance was set at P < 0.05. All statistical analyses were performed using IBM SPSS Statistics (version 25.0; IBM Corp., Armonk, NY, USA).

## Results

### Patient characteristics

A total of 158 female patients with a history of confirmed COVID-19 and hospitalization were included in the study. None of the patients had received COVID-19 vaccines because the vaccines were not yet available for this population at the time of this study. The mean age of the patients was 33.8 ± 6.1 years (range, 21–49 years); 82.3% were married, and most of the patients (66.5%, n = 105) had children. Family member(s) with COVID-19 infection were defined as family member(s) who lived with the patients on a daily basis and established close contact with the patients. A total of 67.1% of patients in this study had family member(s) who had COVID-19 infection. The patient characteristics are summarized in Table 1.

**Table 1:** Patients’ baseline characteristics.

Demographics	
Age, years (mean ± SD)	33.8 ± 6.1
BMI, kg/m^2^ (mean ± SD)	25.9 ± 5.3
Marital status (n [%]) • Single • Married	28 (17.7%) 130 (82.3%)
**Medical history**	
Length of COVID-19 isolation in the hospital, days (mean ± SD)	13.2 ± 6.9
Family member(s) also had COVID-19 infection (n [%]) • Yes • No	106 (67.1%) 52 (32.9%)
Preexisting medical conditions (n [%]) • Fertility disorder • Metabolic disorder • Gastrointestinal disorder • Hematologic disorder • Respiratory disorder • Autoimmune disorder • Oncologic disorder • Cardiovascular disorder • Allergy • No preexisting medical conditions	4 (2.5%) 16 (10.1%) 3 (1.9%) 8 (5.1%) 10 (6.3%) 3 (1.9%) 2 (1.3%) 2 (1.3%) 1 (0.6%) 109 (69%)
No. of pregnancies (n [%]) • 0 • 1 • 2 • 3 • > 3	47 (29.7%) 30 (19.0%) 45 (28.5%) 24 (15.2%) 12 (7.6%)
No. of children (n [%]) • 0 • 1 • 2 • 3 • > 3	53 (33.5%) 33 (20.9%) 45 (28.5%) 20 (12.7%) 7 (4.4%)
Smoking (n [%]) • Yes • No	0 (0%) 158 (100%)
Alcohol consumption (n [%]) • Yes • No	0 (0%) 158 (100%)
Exercise regularity (n [%]) • Less than once per month • 2–4 times per month • More than once per week	65 (41.1%) 29 (18.4%) 64 (40.5%)

Values are presented as the mean ± SD or n (%).

The mean length of hospital isolation was 13.2 ± 6.9 days. Most of the patients had no preexisting medical conditions (69%, n = 109); previous or ongoing metabolic and respiratory disorders were mostly reported by those who had at least one disorder. A total of 67.1% (n = 106) of the patients reported that their family members were also infected with COVID-19 during their hospitalization. The patients’ hospitalization and obstetric histories are summarized in Table 1.

### Menstrual changes

Most patients reported that their cycle length before COVID-19 infection was 24–32 days (79.1%, n = 125). After COVID-19 infection, this group still included the largest number of patients. However, there was an increase in patients with a cycle length of > 32 days or < 24 days. The menstrual pattern changes are summarized in Table 2.

**Table 2:** Menstrual patterns.

Menstrual parameter	Before COVID-19 Infection (n = 158)	After COVID-19 Infection (n = 158)	p value
Mean cycle length (n [%]) • < 24 days • 24–32 days • > 32 days	17 (10.8%) 125 (79.1%) 16 (10.1%)	24 (15.2%) 103 (75.1%) 31 (19.6%)	0.001*
Menstrual irregularity (n [%])	28 (17.7%)	56 (35.4%)	< 0.001*
Heavy menstrual bleeding (n [%])	43 (27.2%)	53 (33.5%)	0.041*
Dysmenorrhea (n [%])	65 (41.1%)	89 (56.3%)	0.454*
Cycle length change (n [%]) • Shortened • Lengthened • None		10 (6.3%) 18 (11.4%) 130 (82.3%)	
Overall menstrual change (n [%])		59 (37.3%)	

* P value results were determined with McNemar’s test. A P value < 0.05 was considered significant.

Compared to the number of patients with menstrual irregularities before (17.7%, n = 28) COVID-19 infection, the number after (35.4%, n = 56) COVID-19 infection doubled (p < 0.001). New cases of HMB after COVID-19 infection were reported by 6.3% (n = 10) of the patients (p = 0.041). No significant changes were found in patients with dysmenorrhea after COVID-19 infection. Overall, 37.3% (n = 59) of the patients reported menstrual pattern changes after COVID-19 infection (Table 2).

### Mental health profiles

The SRQ-29 was used to screen for symptoms of mental health disorders. At least one mental health disorder was noted in 52.5% (n = 83) of the patients, including 32.3% (n = 51) with neurotic symptoms, 12.7% (n = 20) with psychotic symptoms, and 38.0% (n = 60) with PTSD symptoms (Table 3). Patients who screened positive for symptoms of mental health disorders reported higher rates of menstrual irregularity (62.8% vs. 48.7%, p = 0.114), HMB (54.2% vs. 49.1%, p = 0.534), and dysmenorrhea (70.0% vs. 51.4%, p = 0.253), although the difference was not statistically significant. They were also twice as likely to report an overall menstrual change after COVID-19 infection (OR 2.17, 95% CI 1.12–4.22; p = 0.021). Bivariate analysis was conducted to determine the correlation between overall menstrual changes and variables in baseline characteristics (age, BMI, length of isolation, marital status, and preexisting medical conditions). The result showed a significant association between length of isolation and menstrual changes despite the association being weak [r(156) = 0.21, p = 0.009]. Multivariate logistic regression was conducted to analyze possible risk factors for overall menstrual changes using the baseline characteristics (age, BMI, length of isolation, marital status, and preexisting medical conditions). The results showed that a variable length of isolation significantly affected menstrual changes (OR 1.06, 95% CI 1.01–1.12; p = 0.013). However, the results for age (OR 0.96, 95% CI 0.91–1.02; p = 0.259), marital status (OR 0.90, 95% CI 0.39–2.09; p = 0.815), BMI (OR 0.99, 95% CI 0.93–1.05; p = 0.800), and preexisting medical conditions (OR 0.81, 95% CI 0.58–1.13; p = 0.232) were not statistically significant.

**Table 3:** Mental health profiles based on SRQ-29 scores.

Mental health symptoms	Number (percentage)
Neurotic symptoms (n [%]) • Yes • No	51 (32.3%) 107 (67.7%)
Psychotic symptoms (n [%]) • Yes • No	20 (12.7%) 138 (87.3%)
PTSD symptoms (n [%]) • Yes • No	60 (38.0%) 98 (62.0%)

Values are presented as n (%).

## Discussion

SARS-CoV-2 is presumed to affect female fertility through its interaction with angiotensin-converting enzyme 2 (ACE2) [24]. ACE2 is highly expressed in the ovaries, regulating follicular development and ovulation and luteal angiogenesis and affecting regular changes in endometrial tissue. Thus, ACE2 plays a significant role in female reproduction [25]. SARS-CoV-2 may affect female fertility by damaging ovarian tissue and granulosa cells [5]. Moreover, COVID-19 infection is usually accompanied by high levels of interleukin-6, interleukin-8, tumor necrosis factor-α, and other cytokines, which promote a procoagulation state that may affect fetal implantation and development [26].

In our study, we found that the majority of COVID-19 female patients reported menstrual changes after hospitalization and isolation. These changes are associated with the duration of isolation, hospitalization, and signs of mental health problems. A similar study conducted by Li et al. reported that the presence of complications and severe illness were associated with a prolonged menstrual cycle [27]. ACE-2 receptors are also expressed in the ovaries; therefore, SARS-CoV-2 may directly impact menstrual hormone production. Moreover, severe illness caused by infection, including COVID-19 infection, could cause hypothalamic hypogonadism that leads to amenorrhea and infrequent menstruation [16]. This could impact menstrual cycle length due to abnormal hormonal patterns of FSH, LH, estradiol, and progesterone, which all regulate menstrual patterns. A short cycle was associated with an earlier rise in FSH and higher estradiol levels, whereas a longer cycle was associated with higher LH and lower estradiol levels [28]. Our study did not screen for severity or complications. However, from the length of isolation/hospitalization days, we could infer that longer stays in isolation rooms indicated more severe symptoms of COVID-19 [29].

We found that patients who screened positive for symptoms of mental health disorders were twice as likely to report an overall menstrual change after COVID-19 infection. Patients with each type of mental health disorder screened by the SRQ-29 were more likely to report symptoms of menstrual disturbances, namely, neurotic, psychotic, and PTSD symptoms.

Similar findings were also reported by Phelan et al., who found that 46% of the women in their study had experienced menstrual cycle changes since the beginning of the pandemic. In another study, as many as 84% of the women reported experiencing at least one symptom of a mental health disorder, with low mood, anxiety, and poor sleep being the most prevalent symptoms [30]. In Indonesia, a nationwide survey reported similar findings, with 48% of the women reporting psychological distress and 31.6% reporting menstrual changes [31]. However, these studies included women in the general population and studied the effects of the pandemic itself and the social restriction policy. Our study specifically included patients hospitalized due to COVID-19.

Nevertheless, due to the study design, we could not rule out whether the menstrual changes in our participants were due to mental health conditions or physiological changes caused by COVID-19 infection. However, it is clear from our findings that menstrual changes and increased symptoms of mental health disorders do occur in hospitalized female COVID-19 patients. Therefore, providing care regarding these aspects for female patients with COVID-19 is paramount to ensuring more holistic treatment. To restore a healthy menstrual cycle, lifestyle modifications, such as dietary intervention and physical activity, could be the first-line therapy. A study showed that 9 months of a nutritional intervention could restore the menstrual cycle in female athletes with menstrual disorders [32]. Daily physical activity could also help maintain body weight and increase insulin sensitivity, therefore helping regulate the menstrual cycle [33]. Furthermore, to overcome stress and mental health issues caused by the COVID-19 pandemic, there are several positive coping mechanisms that can be used, such as spending time with family members, engaging in different healthy exercises and sports activities, following a routine, and taking a break from social media [34].

This study had several limitations. First, a relatively small number of patients were enrolled. Second, the questionnaire was susceptible to recall bias. However, according to a study by Sampson and Prescott, self-rating is usually the most feasible option and could be used to assess symptoms of premenstrual as well as menstrual patterns [35]. Third, the mental health status of the patients prior to COVID-19 isolation was unknown. Finally, sampling bias may have occurred, as the patients who were able to fill out the survey were mild to moderately ill, while those who were severely ill were unable fill out the survey. Despite these limitations, we believe that digital surveys are suitable and safe for conducting reproductive health research with COVID-19 patients.

## Conclusion

COVID-19 infection and subsequent isolation treatment in hospitalized female patients have an impact on menstrual patterns and mental health. Menstrual changes and increased symptoms of mental health disorders do occur in hospitalized female COVID-19 patients. The length of isolation was the key factor affecting overall menstrual changes and mental health in hospitalized female COVID-19 patients.

Health care providers who treat COVID-19 patients cannot ignore these health problems. Further research employing more clinical examinations and tests is needed to determine the causal relationship between COVID-19 infection and women’s reproductive health.

## Supporting information

S1 Appendix Research questionnaire. (DOCX)

S1 Dataset Raw data. (XLSX)

S2 Dataset Analysis of patients’ characteristic results. (DOCX)

S3 Dataset Analysis of menstrual pattern results. (DOCX)

S4 Dataset Analysis of mental health profile results. (DOCX)

S5 Dataset Analysis of multivariate logistic regression. (DOCX)
