# Supplementary material for: Menstrual cycle changes and mental health states of women hospitalized due to COVID-19
Source: PLoS One. 2022 Jun 24;17(6):e0270658. doi: 10.1371/journal.pone.0270658 (PMC9231764; doi:10.1371/journal.pone.0270658)
Supplement: S1 Appendix — (DOCX) [file pone.0270658.s001.docx]

**Appendix**

**Questionnaire**

**Demographic Profile**

Name (Initial) :

Age : (years)

Marital Status :

Phone Number :

Occupation :

Height : (cm)

Weight : (kg)

BMI :

Number of previous pregnancies :

Number of children :

Pre-existing medical condition :

**Lifestyle**

Do you consume alcohol? YES/NO

Do you smoking? YES/NO

How often do you exercise?

1. Less than once per month
2. 2 – 4 times per month
3. More than once per week

**COVID-19 Status**

Date of COVID-19 diagnosis:

How long have you been isolated in this hospital? (days)

Is there any of your family member that infected by COVID-19? YES/NO

**Menstrual Patterns**

**Date of Day – 1 of last Menstuation:______________________**

**End date of last menstruation:___________________________**

1. How long (in days) in average is the period between your first day of menstrual cycle and the first day of your next menstrual cycle (not including period of spotting outside of your menstrual period)?:

☐ <24 days

☐ 24-28 days

☐ 28-32 days

☐ >32 days

1. How regular is your menstrual cycle?

☐ Regular

☐ Irregular

1. How regular is your menstrual cycle? In other words, how long (in days) in average is the typical length variation between two menstrual cycles?:

☐ <2 days difference

☐ 3-5 days difference

☐ >5 days difference

1. Have you ever not having your period for ≥ 3 months?

☐ If Yes, for how long?

☐ No

1. How many days of bleeding during each menstrual cycle?

☐ <3 days

☐ 3-5 days

☐ >5 days

1. How much blood do you lose on your period?
2. ¼ menstrual pad
3. 1/2 menstrual pad
4. Whole menstrual pad


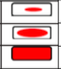


1. Do you have pain during your period?

☐ If yes, could you rate your pain (1-10, 1 is mild pain, 10 is severe pain)

☐ No

**SELF-REPORTING QUESTIONNAIRE - 29 (SRQ-29)**

The following questions are related to certain pains and problems, that may have bothered you in the last 30 days. If you think the question applies to you and you had to describe the problem in the last 30 days, answer YES. On the other hand, if the question does not apply to you and you did not have the problem in the last 30 days, answer NO.

| No | Questions | YES | NO |
| --- | --- | --- | --- |
| SRQ 1 | Do you often have headaches? |  |  |
| SRQ 2 | Is your appetite poor? |  |  |
| SRQ 3 | Do you sleep badly? |  |  |
| SRQ 4 | Are you easily frightened? |  |  |
| SRQ 5 | Do your hands shake? |  |  |
| SRQ 6 | Do you feel nervous, tense, or worried? |  |  |
| SRQ 7 | Is your digestion poor? |  |  |
| SRQ 8 | Do you have trouble thinking clearly? |  |  |
| SRQ 9 | Do you feel unhappy? |  |  |
| SRQ 10 | Do you cry more than usual? |  |  |
| SRQ 11 | Do you find it difficult to enjoy your daily activities? |  |  |
| SRQ 12 | Do you find it difficult to make decisions? |  |  |
| SRQ 13 | Is your daily work suffering? |  |  |
| SRQ 14 | Are you unable to play a useful part in life? |  |  |
| SRQ 15 | Have you lost interest in things? |  |  |
| SRQ 16 | Do you feel that you are a worthless person? |  |  |
| SRQ 17 | Has the thought of ending your life been on your mind? |  |  |
| SRQ 18 | Do you feel tired all the time? |  |  |
| SRQ 19 | Do you have uncomfortable feelings in your stomach? |  |  |
| SRQ 20 | Are you easily tired? |  |  |
| SRQ 21 | Do you drink more alcohol, or do you do drugs? |  |  |
| SRQ 22 | Do you have the thought that someone will harm you in a way? |  |  |
| SRQ 23 | Have disturbing or unusual thoughts been on your mind? |  |  |
| SRQ 24 | Have you ever hallucinate of hearing a sound? |  |  |
| SRQ 25 | Have you had disturbing dreams about traumatic experience, or feel that you experience that situation over again? |  |  |
| SRQ 26 | Are you avoiding activities, places, or people that remind you of that traumatic experience? |  |  |
| SRQ 27 | Do you lose interest in meeting friends or activities that you usually do? |  |  |
| SRQ 28 | Do you feel disturbed if you are in the situation that reminds you of your traumatic experience or when you just thinking about the experience? |  |  |
| SRQ 29 | Do you find it difficult to express your feelings? |  |  |
