# Supplementary material for: Menstrual cycle changes and mental health states of women hospitalized due to COVID-19
Source: PLoS One. 2022 Jun 24;17(6):e0270658. doi: 10.1371/journal.pone.0270658 (PMC9231764; doi:10.1371/journal.pone.0270658)
Supplement: S2 Dataset — (DOCX) [file pone.0270658.s003.docx]

**Table 1. Patients' baseline characteristics**

| **Demographics** | |
| --- | --- |
| Age, years (mean ± SD) | 33.8 ± 6.1 |
| BMI, kg/m^2^ (mean ± SD) | 25.9 ± 5.3 |
| Marital status (n [%])   - Single - Married | 28 (17.7%)  130 (82.3%) |
| **Medical history** | |
| Length of COVID-19 isolation in hospital, days (mean ± SD) | 13.2 ± 6.9 |
| Family member(s) also have COVID-19 infection (n [%]   - Yes - No | 106 (67.1%)  52 (32.9%) |
| Pre-existing medical conditions (n [%])   - Fertility disorder - Metabolic disorder - Gastrointestinal disorder - Hematologic disorder - Respiratory disorder - Autoimmune disorder - Oncologic disorder - Cardiovascular disorder - Allergy - No pre-existing medical conditions | 4 (2.5%)  16 (10.1%)  3 (1.9%)  8 (5.1%)  10 (6.3%)  3 (1.9%)  2 (1.3%)  2 (1.3%)  1 (0.6%)  109 (69%) |
| No. of pregnancies (n [%])   - 0 - 1 - 2 - 3 - > 3 | 47 (29.7%)  30 (19.0%)  45 (28.5%)  24 (15.2%)  12 (7.6%) |
| No. of children (n [%])   - 0 - 1 - 2 - 3 - > 3 | 53 (33.5%)  33 (20.9%)  45 (28.5%)  20 (12.7%)  7 (4.4%) |
| Smoking (n [%])   - Yes - No | 0 (0%)  158 (100%) |
| Alcohol consumption (n [%])   - Yes - No | 0 (0%)  158 (100%) |
| Exercise regularity (n [%])   - Less than once per month - 2–4 times per month - More than once per week | 65 (41.1%)  29 (18.4%)  64 (40.5%) |

Numerical data are presented as mean ± SD. Categorical data are presented as n (%)
