# Supplementary material for: Menstrual cycle changes and mental health states of women hospitalized due to COVID-19
Source: PLoS One. 2022 Jun 24;17(6):e0270658. doi: 10.1371/journal.pone.0270658 (PMC9231764; doi:10.1371/journal.pone.0270658)
Supplement: S3 Dataset — (DOCX) [file pone.0270658.s004.docx]

**Table 2. Menstrual patterns**

| **Menstrual parameter** | **Before COVID-19 Infection (n = 158)** | **After COVID-19 Infection (n = 158)** | **p-value** |
| --- | --- | --- | --- |
| Mean cycle length (n [%])   - < 24 days - 24–32 days - > 32 days | 17 (10.8%)  125 (79.1%)  16 (10.1%) | 24 (15.2%)  103 (75.1%)  31 (19.6%) | 0.001* |
| Menstrual irregularity (n [%]) | 28 (17.7%) | 56 (35.4%) | < 0.001* |
| Heavy menstrual bleeding  (n [%]) | 43 (27.2%) | 53 (33.5%) | 0.041* |
| Dysmenorrhea (n [%]) | 65 (41.1%) | 89 (56.3%) | 0.454* |
| Cycle length change (n [%])   - Shortened - Lengthened - None |  | 10 (6.3%)  18 (11.4%)  130 (82.3%) |  |
| Overall menstrual change (n [%]) |  | 59 (37.3%) |  |

***** P-value results were determined with McNemar's test because the data are paired categorical data. McNemar’s test were conducted to determine differences between variables pre and post COVID-19. P-value < 0.05 is considered significant
