# Supplementary material for: Menstrual cycle changes and mental health states of women hospitalized due to COVID-19
Source: PLoS One. 2022 Jun 24;17(6):e0270658. doi: 10.1371/journal.pone.0270658 (PMC9231764; doi:10.1371/journal.pone.0270658)
Supplement: S4 Dataset — (DOCX) [file pone.0270658.s005.docx]

**Table 3. Mental health profile based on SRQ-29**

| **Mental health symptoms** | **Number (percentage)** |
| --- | --- |
| Neurotic symptoms (n [%])   - Yes - No | 51 (32.3%)  107 (67.7%) |
| Psychotic symptoms (n [%])   - Yes - No | 20 (12.7%)  138 (87.3%) |
| PTSD symptoms (n [%])   - Yes - No | 60 (38.0%)  98 (62.0%) |

Categorical data are presented as n (%)
