# Supplementary material for: Menstrual cycle changes and mental health states of women hospitalized due to COVID-19
Source: PLoS One. 2022 Jun 24;17(6):e0270658. doi: 10.1371/journal.pone.0270658 (PMC9231764; doi:10.1371/journal.pone.0270658)
Supplement: S5 Dataset — (DOCX) [file pone.0270658.s006.docx]

Multivariate Logistic Regression Analysis for Overall Menstrual Changes

| **Variables** | **B** | **S.E Coef** | **OR** | **P-Value** | **95% CI** | |
| --- | --- | --- | --- | --- | --- | --- |
|  |  |  |  |  | **Lower** | **Upper** |
| Age | -0.031 | 0.028 | 0.969 | 0.259 | 0.918 | 1.023 |
| BMI | -0.008 | 0.031 | 0.992 | 0.800 | 0.933 | 1.055 |
| Marital Status | -0.100 | 0.428 | 0.905 | 0.815 | 0.391 | 2.091 |
| Pre-existing medical condition | -0.202 | 0.169 | 0.817 | 0.232 | 0.586 | 1.138 |
| Length of isolation | 0.063 | 0.025 | 1.066 | 0.013* | 1.014 | 1.120 |

Multivariate logistic regression was conducted to analyze possible risk factors for overall menstrual changes using the baseline characteristics (age, BMI, length of isolation, marital status, and pre-existing medical conditions).

P value < 0.05 shows significant correlation
